# Electrical Stimulation Induces Activation of Mitochondrial Apoptotic Pathway and Down-Regulates Heat Shock Proteins in Pork: An Innovative Strategy for Enhancing the Ripening Process and Quality of Dry-Cured Loin Ham

**DOI:** 10.3390/foods13111717

**Published:** 2024-05-30

**Authors:** Xi Yue, Shenghui Bi, Xiangrui Li, Xinxin Zhang, Lisha Lan, Li Chen, Zhili Zhang, Yuanyuan Liu, Ying Zhou, Chun Ye, Qiujin Zhu

**Affiliations:** 1 School of Liquor and Food Engineering, Guizhou University, Guiyang 550025, China; 17585519813@163.com (X.Y.); 1215331352bzh@163.com (S.B.); 18212437705@163.com (X.L.); zx2410197583@163.com (X.Z.); 18230853455@163.com (L.L.); cl199901062543@163.com (L.C.); 18311600408@163.com (Z.Z.); yyliu3@gzu.edu.cn (Y.L.); zhying_0525@163.com (Y.Z.); yechun126@163.com (C.Y.); 2 Key Laboratory of Agricultural and Animal Products Storage and Processing, Guizhou University, Guiyang 550025, China

**Keywords:** ham ripening, electrical stimulation, mitochondria, myofibril degradation, apoptosis, heat shock proteins

## Abstract

A fundamental regulatory framework to elucidate the role of electrical stimulation (ES) in reducing long production cycles, enhancing protein utilization, and boosting product quality of dry-cured ham is essential. However, how mitochondria and enzymes in meat fibers are altered by ES during post-processing, curing, and fermentation procedures remains elusive. This study sought to explore the impact of ES on the regulation of heat shock proteins (HSP27, HSP70), apoptotic pathways, and subsequent influences on dry-cured pork loin quality. The gathered data validated the hypothesis that ES notably escalates mitochondrial oxidative stress and accelerates mitochondrial degradation along the ripening process. The proapoptotic response in ES-treated samples was increased by 120.7%, with a cellular apoptosis rate 5-fold higher than that in control samples. This mitochondrial degradation is marked by increased ratios of Bax/Bcl-2 protein along the time course, indicating that apoptosis could contribute to the dry-cured ham processing. ES was shown to further down-regulate HSP27 and HSP70, establishing a direct correlation with the activation of mitochondrial apoptosis pathways, accompanied by dry-cured ham quality improvements. The findings show that ES plays a crucial role in facilitating the ripening of dry-cured ham by inducing mitochondrial apoptosis to reduce HSP expression. This knowledge not only explains the fundamental mechanisms behind myofibril degradation in dry-cured ham production but also offers a promising approach to enhance quality and consistency.

## 1. Introduction

Dry-cured meat is esteemed as a culinary treasure within the realm of meat products, skillfully produced through a series of meticulous processes such as curing, drying, and ripening. These products are revered for their distinctive color, flavor, and sensory attributes, garnering extensive admiration from consumers worldwide [1]. Despite their popularity, the traditional methods of dry-cured meat production are often marred by prolonged processing durations, leading to inconsistencies in product quality and presenting formidable obstacles in achieving reliable operational management. In this era, where consumers exhibit an increasingly refined palate, there is a growing demand for meat products that excel not only in taste but also in terms of safety, nutritional value, and health benefits.

In the recent past, a variety of innovative technologies have captured significant interest in the spheres of meat science and processing. Prominent among these are pulsed electric fields (PEF), ultrasonic (US), and high–pressure processing (HPP). Within this spectrum, electrical stimulation (ES) technology distinguishes itself as capable of inducing desired reactions by applying pulsed currents [2]. Endowed with attributes such as energy efficiency, environmental sustainability, and economic viability, ES has been increasingly embraced by the cattle and sheep slaughtering industries due to its straightforward application and remarkable effectiveness [3]. Empirical evidence suggests that ES accelerates glycolysis within the muscle tissues post-slaughter, culminating in a swift reduction in pH levels and an expedited onset of rigor mortis [4]. The ripening of muscle tissue post-slaughter is predominantly contingent upon the activation of intrinsic enzymatic systems, with ES creating an optimal milieu for the activation of calpain enzymes within the carcass [5]. This surge in enzymatic activity fosters the degradation of proteins, resulting in the release of a more substantial quantity of free amino acids, thereby reducing the aging period and augmenting the meat’s flavor [6]. In essence, ES has proven its efficacy in enhancing the quality of muscle tissue after slaughter, manifesting improvements in characteristics such as color, tenderness, water retention, and flavor. These enhancements can be attributed to the technology’s impact on the physiological and biochemical processes within the carcass, which subsequently modifies the muscle microstructure and the structural and functional properties of proteins [7].

Although apoptosis of muscle cells occurs during slaughter [8], it should be seen as a continuous process that extends into late processing and overlaps with cell death. Furthermore, there is a noticeable lack of research on how processing, curing, and fermentation alter mitochondria and enzymes in meat fibers. Our previous study confirmed that ES still emerges as an innovative and safe method for controlling the residual endogenous biomass of apoptotic cells to participate in the optimization of meat quality. ES plays a crucial role in ensuring the health and safety of dry-cured meat. Following a 120 V, 50 s ES treatment, the content of N-Glycolylneuraminic acid (Nue5Gc) in red meat can be reduced by 74.24 ± 0.69%, with negligible effects on the meat’s texture and color [9]. The application of ES in the processing of dry salted meat highlights its intricate interplay with cathepsin activity and its consequential impact on protein degradation, thereby enhancing the product’s quality and taste characteristics [10]. Our investigation explores the potential of secondary excitation effects within the field of ES, providing key insights that are highly beneficial to research activities in meat science. The results of this study highlight the considerable promise that ES holds for enhancing the production quality of premium fermented meats.

Throughout the maturation and fermentation journey, dry-cured meat experiences a plethora of physical and chemical metamorphoses, encompassing water loss, textural modification, and the proteolysis and lipolysis of muscle constituents. These transformative events are instrumental in sculpting the quintessential attributes of dry-cured meat [11]. The proteolytic breakdown of myofibrillar proteins by indigenous proteases yields an array of smaller peptides, positioning dry-cured meat as a natural repository of bioactive peptides. This aspect presents a rich avenue for delving into the nutritional profiles of food products.

Moreover, the incursion of proteomics into the realm of meat quality assessment has unveiled a multitude of heat shock proteins (HSPs) present within muscle tissues. These proteins play a pivotal role in the modulation of cellular apoptosis pathways, particularly at the mitochondrial level [12]. They exert a profound influence on the degradation of muscle proteins and the evolution of quality characteristics, thus being recognized as significant regulators during the meat ripening process [13]. Contreras observed that post-slaughter, low-pressure ES intervention augmented the hydrolysis of HSP20, culminating in a significant enhancement of beef tenderness [14]. However, a gap exists in the literature concerning the progressive modulation of these intrinsic biomaterials by extending the application of ES into the meat product processing stage, as well as an exploration of the underlying mechanisms.

Considering the dearth of research on mitochondrial apoptosis in muscle cells, alterations in heat shock proteins, and their subsequent impact on the quality of dry-cured meat post-ES treatment, this study embarks on an exhaustive examination of the influence exerted by ES on the curing process and the ensuing quality modifications in dry-cured meat. The investigation is designed to meticulously scrutinize the alterations in cellular responses within muscle fibers, the expressional shifts of proteins HSP27 and HSP70, and the contribution of mitochondrial apoptosis to the meat’s ripening trajectory after ES application.

## 2. Materials and Methods

### 2.1. Samples Preparation of Fresh Meat and Cured Meat

The pigs utilized in this study were procured from Guizhou Tainong Xingwang Food Co., Ltd. (Guiyang, China). Their diet predominantly consisted of corn, sorghum, and soybeans. Upon achieving an approximate live weight of 250 kg and after a growth period exceeding 365 days, the animals were humanely euthanized using electric stunning by the breeder. Subsequently, the pigs were transported to the slaughterhouse in a truck. At the slaughterhouse, the pigs underwent a series of processes including showering, slaughtering, and trimming. These procedures were meticulously conducted to ensure the hygiene, safety, and quality of the pork. After these processes, pork loins were meticulously selected, immediately refrigerated, and expeditiously transported to the laboratory within a 12 h window following slaughter.

Fresh pork tenderloins, each with an approximate mass of 500 g, were randomly assigned to two groups. The treatment group was subjected to electrostimulation using an AC stable power supply (Epps Power Supply Co., Ltd., Suzhou, China), which was set to a current intensity of 0.6 A for 60 s. The non-stimulation group (NS, 0 A) served as the control. Throughout the electrostimulation process, the temperature fluctuations of the pork were closely monitored using a thermal infrared imager (North Embed Technology Dalian Co., Ltd., Dalian, China).

Divergent from previous studies, cured meat samples were established in this research. Both the ES and NS groups were subjected to the dry curing method, employing a uniform salt concentration of 3% relative to the mass of the samples. The samples were then mixed with salt, vacuum-packed, and cured for 5 days at a temperature of 4 °C. After the curing phase, the samples underwent a drying process for 7 days at 8 °C under a relative humidity of 80%. Eventually, the samples were ripened for 20 days at a temperature of 15 °C with the RH varying from 65% to 85%. The dry-cured pork loins were obtained upon the completion of 30 days of processing. Samples, with triplicate sets for each time point, were collected on days 1, 5, 12, 20, and 30 of the processing period.

### 2.2. Measurement of Quality Indicators

The pH value of the samples was measured using a digital pH meter (PHS-3C, Shanghai Yueping Scientific Instruments Co., Shanghai, China). A precise quantity of minced meat sample, 1 g, was weighed and subsequently diluted with an equal volume of ultrapure water. The mixture was then homogenized for 60 s at a rotation speed of 2800 r/min using an XHF-D homogenizer (Ningbo Xinzhi Biotechnology Co., Zhejiang, China). Before the measurement, the pH meter was meticulously calibrated with standard buffers at pH values of 4.01 and 6.86 at an ambient temperature of 22 °C.

For the texture profile analysis (TPA), samples were trimmed into cubic pieces of dimensions 30 mm × 30 mm × 30 mm, aligned with the direction of the muscle fibers. The TPA was conducted using a TOUCH texture analyzer (Bao Sheng Technology Co., Ltd., Shanghai, China). The analyzer was set with the following parameters: a pre-test speed of 2 mm/s, a test speed of 0.5 mm/s, a post-test speed of 0.5 mm/s, a target value set at 5.0 mm, and a probe type of TA4/1000 [15].

Samples were further processed into uniform strips with dimensions of 20 mm × 20 mm × 20 mm. These strips were subjected to shear testing along an axis perpendicular to the muscle fibers. A muscle tenderizer (model C-LM3B, Northeast Agricultural University, Harbin, China) was employed to measure the shear force, maintaining a shear speed not exceeding 5 mm/s [16].

A 3 g sample was weighed and combined with 30 mL of myofibrillar fragmentation index (MFI) extraction solution prepared with the following composition: 100 mM KCl, 7 mM KH_2_PO_4_, 18 mM K_2_HPO_4_, 1 mM EDTA, and 1 mM MgCl_2_, adjusted to a pH of 7.0, and maintained at a temperature of 2 °C. The samples were homogenized for 60 s and then centrifuged at 10,000× *g* for 15 min at 4 °C. The supernatant was discarded, leaving the precipitate. This procedure was repeated thrice. The final precipitate was dissolved in 15 mL of extraction solution at 2 °C, filtered through a 150-mesh gauze, and the protein concentration of the filtrate was determined using the BCA method. The filtrate’s concentration was adjusted to a standard protein concentration of 0.5 mg/mL using the MFI extraction solution. The absorbance was then measured at 540 nm. The mean of three absorbance readings was calculated as the MFI value. Finally, this mean value was multiplied by 200 to obtain the MFI [17].

### 2.3. Measurement of HSP27 and HSP70 Expression

Proteins were extracted from the samples by the protocol provided with the ELISA kit (Solarbio Science & Technology Co., Ltd., Beijing, China). Each sample was accurately weighed, and a 150 mg aliquot was finely ground using a mortar and pestle. To each sample, 1.5 mL of RIPA tissue lysate and 15 μL of PMSF (Phenylmethylsulfonyl fluoride) were added to facilitate thorough grinding. The resulting homogenate was subjected to centrifugation at 10,000× *g* for 20 min at 4 °C. The supernatant, which was collected post-centrifugation, contains the heat shock proteins and was subsequently stored at −80 °C for future analysis.

Prior to the commencement of the assay, the ELISA kit was retrieved from the 4 °C refrigerator and allowed to equilibrate to room temperature (20 °C) for a duration of 30 min. A linear regression curve was constructed for the standard, ensuring a coefficient of R^2^ greater than or equal to 0.990. This curve was plotted with the mass concentrations of the standard solutions of varying gradients along the *x*-axis, and the corresponding OD values measured at 450 nm on the *y*-axis. Utilizing the curve equation, the protein concentrations of the samples were calculated.

### 2.4. Mitochondrial Extraction

The samples were finely minced and subsequently immersed in a mitochondrial separation medium, prepared at 10 times the volume of the sample. The composition of the medium included 230 mM mannitol, 70 mM sucrose, and 2 mM EDTA, adjusted to a pH of 7.4. The homogenate was subjected to centrifugation at 1000× *g* for an initial period of 10 min at a temperature of 4 °C. This centrifugation step was repeated twice under identical conditions to ensure thorough separation. Following these steps, the supernatant was further centrifuged at 8000× *g* for 20 min to precipitate the mitochondria [18]. The resulting pellet constituted the mitochondrial fraction, while the supernatant contained the cytoplasmic components. The protein concentration of both fractions was quantified using the BCA assay method.

### 2.5. Measurement of Mitochondrial Structure Indicators

A diluted mitochondrial solution, prepared to a concentration of 0.5 mg/mL, was aliquoted (300 µL) and combined with 2700 µL of a test medium. This medium contained 230 mM mannitol, 70 mM sucrose, and 3 mM Hepes, buffered to a pH of 7.4. The mixture was incubated at 25 °C for 3 min. Subsequently, the mitochondrial membrane permeability was assessed by measuring the absorbance at 540 nm [19].

The mitochondrial protein concentration was adjusted to 0.5 mg/mL and incubated with a mixture of specific reagents, comprising 400 µL of 0.5 mM FeSO_4_ and 0.4 mL of 0.5 mM VC (ascorbic acid), at a temperature of 37 °C for 15 min. The extent of mitochondrial swelling was inferred from the absorbance measurements at 520 nm [20].

The mitochondrial protein concentration was diluted to 0.3 mg/mL using a mitochondrial permeability transition pore (MPTP) solution, composed of 230 mM mannitol, 70 mM sucrose, and 3 mM Hepes, adjusted to a pH of 7.4. Specific reagents, including 0.5 mM FeSO_4_ and 0.4 mL of 0.5 mM VC, were combined with 3 mL of the MPTP solution. The mixture’s absorbance was recorded at 540 nm, which reflects the open state of the mitochondrial MPTP [21].

### 2.6. Measurement of Mitochondrial Oxidative Stress

The content of reactive oxygen species (ROS) was determined by first weighing approximately 0.5 g of the sample. The sample was then subjected to homogenization in an ice bath with the addition of 4.5 mL of PBS (0.01 M, pH 7.2). Following homogenization, the mixture was centrifuged at 3000× *g* and 4 °C for 10 min. The supernatant, which contains the ROS, was collected for further analysis using a commercially available assay kit (Yankang Biotechnology Co., Ltd., Shanghai, China).

Superoxide dismutase (SOD) activity was quantified based on the amount of enzyme required to inhibit the reduction of a specific dye by 50% per 1 mg of histone in 1 mL of the reaction mixture. This assay was conducted according to the manufacturer’s instructions provided with the assay kit (Solarbio Science & Technology Co., Ltd., Beijing, China).

The content of malonaldehyde (MDA) was measured by weighing approximately 0.5 g of the sample and mixing it with 5 mL of extraction solution. The mixture was then centrifuged at 8000× *g* and 4 °C for 10 min. The supernatant, which contains MDA, was collected for determination using a standard assay procedure (Solarbio Science & Technology Co., Ltd., Beijing, China).

### 2.7. Measurement of Bcl-2 Family Protein Content

The levels of Bcl-2 and Bax, which are representative proteins of the Bcl-2 family, were quantified using ELISA kits (Solarbio Science & Technology Co., Ltd., Beijing, China). The procedure commenced with the precise weighing of 0.5 g of the sample. Thereafter, 4.5 mL of PBS (10 mM, pH 7.4) was added. The mixture was thoroughly combined and subjected to grinding on ice for 10 min. After grinding, the homogenate was centrifuged at a speed of 10,000× *g* for 15 min at 4 °C to achieve a complete separation. The supernatant, which was enriched with the soluble proteins of interest, was then collected and immediately placed on ice for subsequent assay procedures.

### 2.8. Measurement of Caspase-3 and Caspase-9 Activity

The activity of caspase-3 and caspase-9 was determined by visible photometric colorimetry and reference to the kit instructions. An amount of 0.5 g of meat sample was accurately weighed, and placed in a mortar, and 5 mL of caspase lysate was added to an ice bath Pulp, transfer the homogenate was to a 5 mL centrifuge tube and centrifuged (10,000× *g*, 4 °C, 15 min), take the supernatant and put it away. Place another EP tube on ice to be tested.

### 2.9. Measurement of Microstructure (SEM)

Samples were cut into 2 mm × 2 mm × 5 mm slices (perpendicular to the muscle fibers). The slices were fixed in a 2.5% *v*/*v* solution of glutaraldehyde for 48 h, then dehydrated twice, each using different concentrations of ethanol (25%, 50%, 70%, 95%, and anhydrous ethanol), freeze-dried for 48 h, and then sprayed with gold [22]. Finally, the microstructure of the sample was observed at a magnification of 20 μm.

### 2.10. HE Stain

Samples were cut into 4 μm sections using a microtome. After deaxing of paraffin sections to water, the sections were stained with Harris hematoxylin for 8 min, washed, differentiated with 1% hydrochloride alcohol for several seconds, rinsed, turned blue with 0.6% ammonia, and then washed with running water. Sections were added into the eosin dye solution for 3 min [23]. The sections were dehydrated and transparent, dried, neutral gum sealed, and photographed under a microscope.

### 2.11. Measurement of Apoptosis Rate

Apoptosis during ripening was detected by in situ end (TUNEL) staining. Referring to Cao et al. [24], samples were cut into about 1 cm × 1 cm × 0.5 cm, fixed in 4% paraformaldehyde solution, paraffin sections, placed in a 60 °C incubator, baked slices for 45 min, stained with xylene and 100% ethanol, respectively, immersed in a gradient concentration of ethanol solution (90%, 80%, 70%, and 50%), and then repaired with sodium citrate (pH 6.2) for 5 min. It was permeabilized in solutions containing 0.1% Triton X-100 and 0.1% sodium citrate. Closed with 3% hydrogen peroxide solution, added with 50 μL Tunel reaction buffer, incubated at 37 °C in light for 80 min, stained with DAPI, sealed with an anti-fluorescent extinguishing agent, and then observed and photographed under a fluorescence microscope for a short time, and 3 different fields were calculated under 200 times microscope. The area of apoptotic cells in each field was calculated and analyzed by Image-Pro Plus 6.0 software.

### 2.12. Statistical Analysis

Statistical analysis was conducted using the general linear model program of SPSS software (Version 25.0 SPSS Inc., Chicago, IL, USA). The different treatments, storage time, and their interaction were used as fixed effects, and the carcass was regarded as a random term. The least significant differences (LSD) test was used to identify the significance of the analysis of the differences (*p* < 0.05). The data were reported as the mean ± standard errors (S.E). Graphing used GraphPad Prism software 2022 (GraphPad Software Inc., San Diego, CA, USA) and Origin software 2021 (OriginLab Corporation, Northampton, MA, USA).

## 3. Results and Discussion

### 3.1. Quality Indicators

The pH level of the muscle reflects the overall chemical environment in the cell, which has an important influence on quality indicators such as color, hydraulic strength, and tenderness of the meat. As shown in Table 1, the pH increased in both groups due to the accumulation of alkaline substances from protein degradation during ham ripening [25]. The pH in the ES group was significantly lower than in the NS group (*p* < 0.05), attributed to the spread of ES causing extensive muscle contraction, resulting in increased glycogen consumption and lactate production [26].

Hardness, which is the force required to deform food to a certain extent, reflects the internal binding forces that maintain the food’s structure. Elasticity refers to the ability of a material to deform under an external force and revert to its original shape upon the force’s removal. Chewiness, which is related to both hardness and elasticity, represents the effort expended during the mastication of solid food until it is palatable for swallowing [27]. The analysis shows that the longer the dry-cured pork loin matures, the meat quality becomes compact, the delicacy decreases, and the chewing and taste increase (Table 1). The hardness and chewing values of the ES group were significantly lower than those of the NS group (*p* < 0.05). ES significantly improved the textural characteristics of the product, giving a better taste of dry-cured pork loin, and improving eating comfort.

Shear force is a direct measure of meat tenderness, with lower values indicating a more tender product quality. Over time, an increasing trend in shear force was observed for the dry-cured pork loins in both groups; however, a notable difference in shear force was evident between the treatment groups (Table 1). The ES group consistently displayed significantly lower shear force values than the NS group (*p* < 0.05). It can be concluded that the application of ES significantly expedited the tenderization process and reduced the ripening time during the maturation of the dry-cured pork loin. This effect is likely due to ES accelerating glycolysis and the breakdown of myofibrillar and connective tissues in post-slaughtered meat, simultaneously disrupting the myofibrillar structure, which in turn enhances tenderness [28].

The MFI is a crucial parameter for assessing meat tenderness, reflecting the extent of myofibrillar structure breakdown and protein degradation. Higher MFI values suggest a greater degree of myofibrillar disintegration [29]. Throughout the muscle ripening process, myofibrils are cleaved from the Z-line, leading to their fragmentation. A significant increase in MFI values was observed for both groups over time (*p* < 0.05), with the ES group showing an MFI value 1.4 times higher than that of the NS group on day 5 (Table 1). Related studies have indicated that ES treatment can damage the sarcoplasmic reticulum and accelerate myofibrillar protein degradation, thereby increasing the MFI [30]. Additionally, the high-temperature, acidic environment induced by ES treatment is believed to enhance enzymatic activity, accelerate protein breakdown, and improve meat tenderness [31].

### 3.2. Microstructure

**Figure 1:**
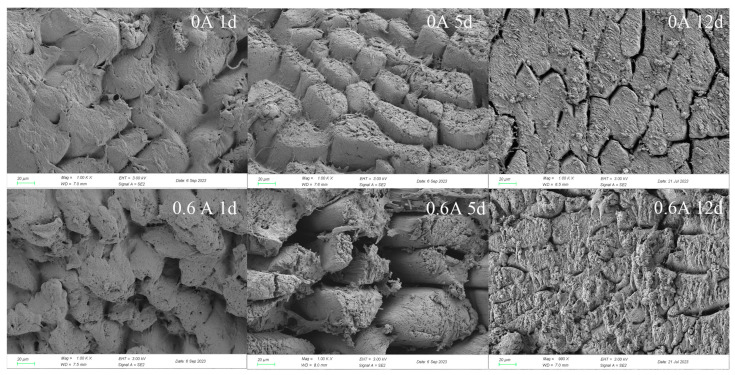
Effect of ES on the microstructure during ripening of dry-cured pork loin.

The microstructure of the dry-cured pork loin significantly changed. In the ES group, muscle fiber bundles were broken and fractured with larger gaps. As the meat matured, the microstructure of muscle fibers in the ES group became loose and disrupted, losing structural integrity and becoming rough, and the fragmentation of muscle fibers is more obvious (Figure 1). In contrast, the NS group maintained a more intact microstructure with a smoother surface. These observations suggest that ES treatment not only altered the arrangement of muscle fibers in the dry-cured pork loin but also inflicted damage upon the muscle fiber structure. Such changes are conducive to enhancing the meat’s tenderness, corroborating the findings of the increased MFI due to ES treatment. The observed microstructural changes are attributed to the ES treatment, which is known to augment the release of calcium ions from the sarcoplasmic reticulum. This leads to the formation of contracture bands and the subsequent disruption of the myofiber structure [32].

In conclusion, the quality assessment of the dry-cured pork loin revealed that the application of ES treatment resulted in the destruction of muscle tissue structure and accelerated the degradation of myofibrils. This treatment significantly reduced the shear force, hardness, and chewing value of the dry-cured pork tenderloin, thereby endowing it with superior taste and tenderness.

### 3.3. Expression Level of HSP27, HSP70

**Figure 2:**
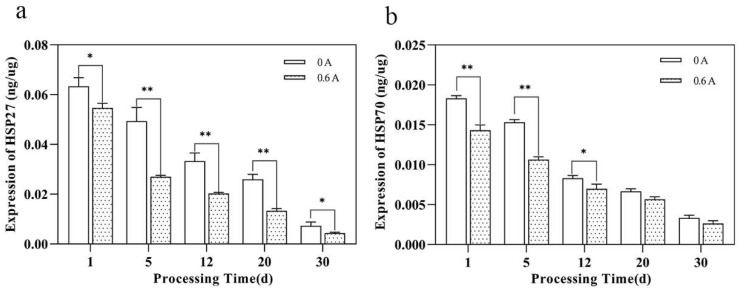
Effect of ES on the expression level of HSPs during ripening of dry-cured pork loin. (**a**) Expression level of HSP27; (**b**) expression level of HSP70; (*, *p* < 0.05; **, *p* < 0.01).

HSP27 is a molecular chaperone that is abundantly expressed in skeletal muscle and closely associated with meat quality [33]. The cessation of external oxygen and nutrient supply inevitably triggers apoptosis. As a stress protein, HSP27 elicits an immediate stress response to counteract apoptosis, aiming to maintain cellular homeostasis, which explains its peak expression levels at day 1. Thereafter, HSP27 assumes the role of a molecular chaperone, binding to damaged and misfolded proteins, thus preventing irreversible damage [34]. The auto-degradation process leads to a gradual decrease in HSP27 expression within the tissues (*p* < 0.05). Notably, the expression of HSP27 in the ES group was significantly lower than that in the NS group (*p* < 0.05). Most markedly, at day 5, HSP27 expression declined by 45.57% relative to the NS group (Figure 2a). Previous studies have established that HSP27 plays a vital role in cellular maintenance and repair within the organism, with functions such as acting as a molecular chaperone, regulating actin polymerization, modulating interactions between intermediate filaments, and inhibiting stress-induced apoptosis, as well as preserving the stability of the myofibrillar structure [35]. The down-regulation of HSP27 expression due to ES treatment in the dry-cured pork loin attenuates its function in maintaining the stability of the myofibrillar structure.

The expression trend of HSP70 mirrored that of HSP27, and there was a higher expression of HSP27 than HSP70 (Figure 2b). HSP70 expression was significantly lower in the ES group compared to the NS group (*p* < 0.05, except days 20 and 30). A 32.46% decrease in HSP70 expression in the ES group, compared to the NS group, indicates that ES treatment leads to the down-regulation of HSP70 expression in the meat. Proteomic studies have correlated HSP70 with meat tenderness, where lower expression levels are associated with reduced shear force [36]. The up-regulation of HSPs in stiffer meat corresponds to the lower shear force value observed in the ES group in this study. The concentrations of these HSPs in the ES muscles at later stages of aging, as compared to the NS muscles, are significantly different from those reported in previous studies [14].

### 3.4. Mitochondrial Structure

**Figure 3:**
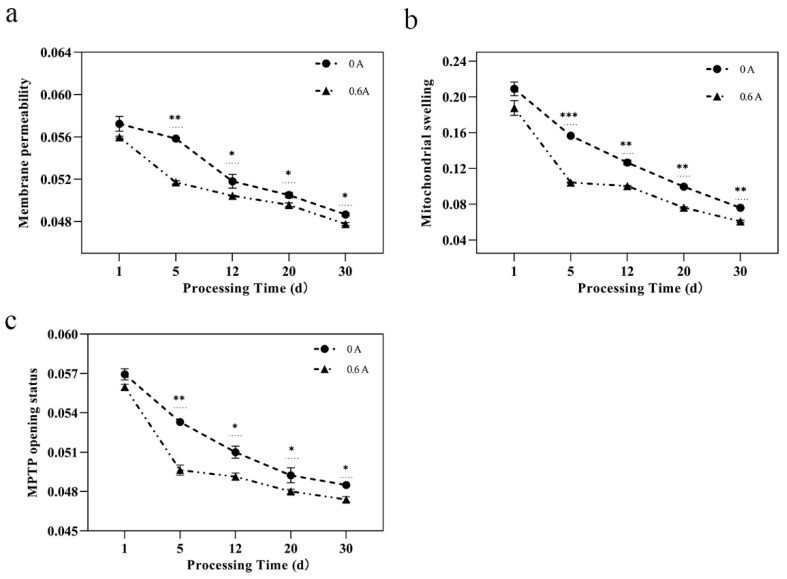
Effect of ES on the mitochondrial function during ripening of dry-cured pork loin. (**a**) Mitochondrial membrane permeability; (**b**) mitochondrial swelling; and (**c**) MPTP opening status; (*, *p* < 0.05; **, *p* < 0.01; and ***, *p* < 0.001).

Mitochondria serve as pivotal regulators of apoptosis, exerting a crucial influence on the apoptotic process. Alterations in mitochondrial membrane permeability are indicative of structural damage to the mitochondria. Although the differences in absorbance values between groups were not significant at day 1 (*p* > 0.05), the ES group displayed a markedly lower value after 5 days (*p* < 0.001) (Figure 3a). Lower absorbance values suggest increased mitochondrial membrane permeability, whereas higher values denote the opposite. ROS can assault lipids and proteins, subsequently impairing the mitochondrial membrane, and leading to damage [37]. The application of ES generates ROS, elevates the mitochondrial bilayer membrane permeability transition pore, and modulates the membrane fluidity. An increase in membrane permeability can directly precipitate rapid shifts in the mitochondrial physiological environment, causing disarray in various metabolic reactions and thus impairing mitochondrial physiological functions [38].

The preservation of mitochondrial morphology is fundamental to the normal functioning of both mitochondria and cells. Similar to the membrane permeability findings, the differences in absorbance values between groups at day 1 were not significant (*p* > 0.05), but the ES group showed a significantly lower value after 5 days (*p* < 0.001) (Figure 3b). The decreasing trend in absorbance values suggests a progressive increase in mitochondrial swelling [39], indicating that ES significantly amplifies this phenomenon. The enhancement of mitochondrial swelling is likely closely related to the disruption of Ca^2+^ homeostasis and the opening of the mitochondrial permeability transition pore (MPTP). A substantial influx of Ca^2+^ from the muscle cytoplasm into the mitochondria can ultimately lead to mitochondrial swelling [40]. Moreover, ES can perturb the normal architecture of the sarcoplasmic reticulum, leading to the release of Ca^2+^. This release may be connected to the observed alterations in mitochondrial structure and function [41].

An increase in MPTP openness can induce changes in the osmotic pressure of matrix proteins, and a decrease in absorbance value reflects an elevated MPTP openness [42]. The absorbance values indicated that ES treatment hastened the irreversible opening of MPTP after 5 days (*p* < 0.01) (Figure 3c). The active mechanism of ES is analogous to PEF treatment, where a current is applied between two electrodes. PEF technology introduces noninvasive changes to the tissue structure, alters cell membrane structure, and enhances mass transfer capabilities by inducing the electroporation phenomenon [43]. It is hypothesized that ES may also induce a similar “electroporation” effect, promoting the irreversible opening of the mitochondrial MPTP.

### 3.5. Mitochondrial Oxidative Stress and Damage

**Figure 4:**
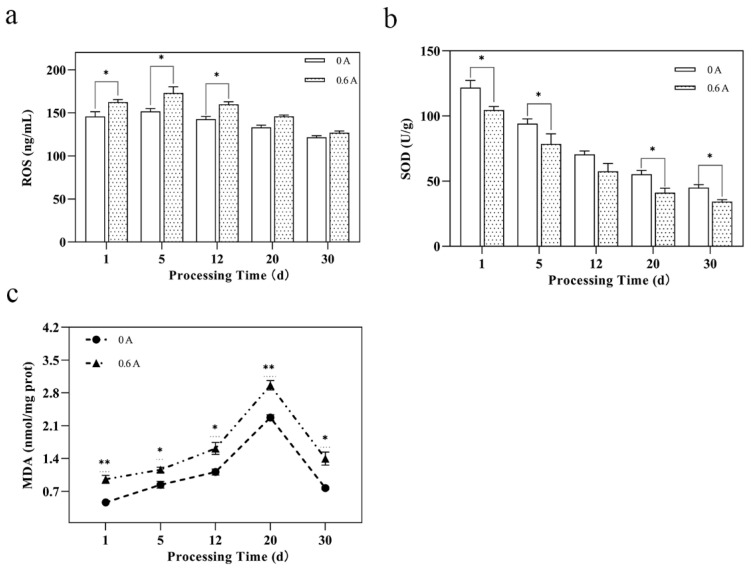
Effect of ES on mitochondrial oxidative stress and damage during ripening of dry-cured pork loin. (**a**) ROS content; (**b**) SOD activity; and (**c**) MDA content; (*, *p* < 0.05; **, *p* < 0.01).

ROS are pivotal signaling molecules generated by mitochondria. A regulated level of ROS is essential for cellular processes such as proliferation and signaling in a physiological state, which has been established as a primary contributor to apoptosis [44]. Between day 1 and day 5, the mitochondrial antioxidant capacity declined gradually, while the ROS levels escalated, reaching a peak at day 5 (Figure 4a). Subsequently, ROS production diminished as numerous muscle cells underwent apoptosis. Concurrently, the ROS content in the ES group was significantly higher than that in the NS group (*p* < 0.05). Excessive ROS accumulation can lead to the oxidation of polyunsaturated fatty acids (PUFAs) in the cell membrane, triggering lipid peroxidation [45]. Additionally, ROS can oxidize unsaturated fatty acids within the mitochondrial membrane, increasing its permeability and reducing membrane fluidity [46]. By days 20 to 30, no significant difference in ROS content was observed between the two groups, indicating that the influence of ES on ROS levels gradually waned.

SOD is a vital intracellular antioxidant enzyme that neutralizes ROS. The reduced SOD activity in the ES group (*p* < 0.05) implies that ES treatment compromised mitochondrial antioxidant defenses, thereby elevating mitochondrial oxidative stress (Figure 4b). MDA, the end product of lipid peroxidation, serves as an indirect indicator of ROS-induced damage, which is intricately linked to the onset of cellular apoptosis. From day 1 to day 20, MDA content showed an increasing trend in both groups due to the disruption of the antioxidant system as the ripening time extended, leading to heightened oxidation. Between days 20 and 30 (Figure 4c), MDA levels decreased, signifying that the degradation of hydrogen peroxide, an initial oxidation byproduct, outpaced its formation. Notably, the ES group exhibited significantly higher MDA levels than the NS group (*p* < 0.01). Consistent with these findings, Biffin et al. [47] reported that ES intensified the degree of lipid oxidation in alpaca muscle. It is recognized that lower MDA levels correlate with less membrane damage, whereas higher levels suggest greater membrane damage, indicating that ES may exert further effects on mitochondrial membrane integrity.

### 3.6. Bcl-2 Family Protein Content

**Figure 5:**
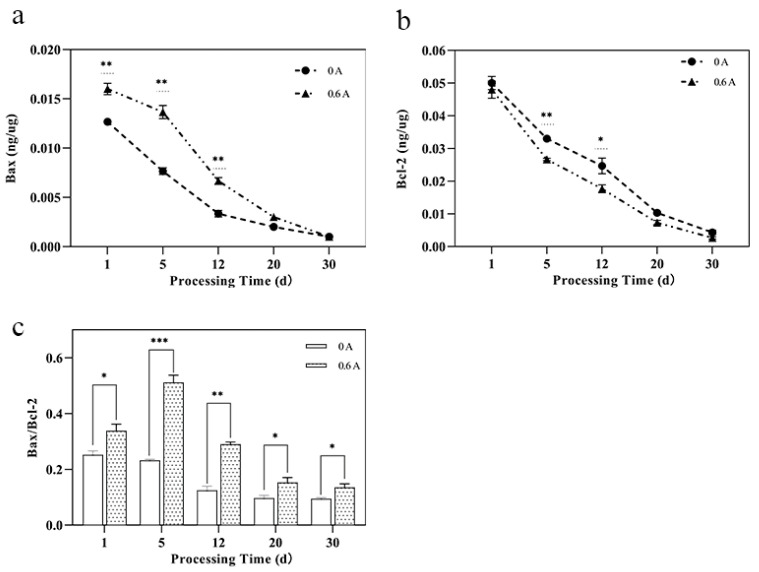
Effect of ES on Bcl-2 family proteins during ripening of dry-cured pork loin. (**a**) Bax content; (**b**) Blc-2 content; and (**c**) Bax/Blc-2; (*, *p* < 0.05; **, *p* < 0.01; and ***, *p* < 0.001).

Bax, a pro-apoptotic protein, accumulates on the outer mitochondrial membrane under conditions that induce apoptosis, thereby triggering the release of pro-apoptotic factors. Conversely, Bcl-2, an anti-apoptotic protein, functions to inhibit Bax activity. The equilibrium between Bax and Bcl-2 is crucial for the regulation of apoptosis [48]. Throughout the ripening process, the levels of both Bax and Bcl-2 proteins decreased, with the highest values observed at day 1 (Figure 5a,b). Consistent with our findings, previous studies have reported that the peak Bax content during chicken meat ripening occurs on day 1, with Bcl-2 content also exhibiting a declining trend. The Bax content in the ES group was significantly higher than in the NS group (*p* < 0.01), whereas the Bcl-2 content in the ES group was significantly lower than that in the NS group (*p* < 0.01). Intracellular stimulation can induce changes in the inner mitochondrial membrane, leading to the opening of the MPTP and a loss of mitochondrial membrane potential. This event results in the release of various pro-apoptotic factors from the intermembrane space of the mitochondria into the cytosol, potentially explaining the increased degradation of Bax protein following ES treatment.

The apoptotic effect was evaluated by calculating the ratio of Bax to Bcl-2. A high Bax/Bcl-2 ratio indicates a greater pro-apoptotic capacity, whereas a low ratio suggests a stronger anti-apoptotic ability. The Bax/Bcl-2 level in the ES group was significantly higher than that in the control group (*p* < 0.001), with a particularly substantial increase observed at day 5, where it was 120.7% higher than that in the NS group (Figure 5c). The data indicate that ES treatment can modulate the levels of Bcl-2 family proteins, accelerate the degradation of caspases by elevating the Bax/Bcl-2 ratio, and thereby induce apoptosis.

### 3.7. Caspase-3 and Caspase-9 Activity

**Figure 6:**
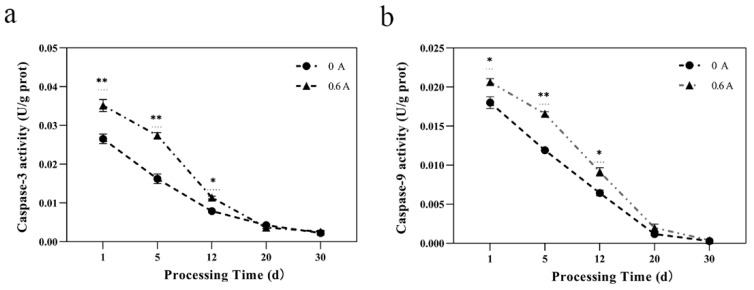
Effect of ES on caspase enzyme activity during ripening of dry-cured pork loin. (**a**) Caspase-3 activity; (**b**) Caspase-9 activity; (*, *p* < 0.05; **, *p* < 0.01).

Caspases play an integral role as enzymes in the apoptotic pathway. Initiator caspases, including caspase-2, -8, -9, and -10, initiate a cascade that activates downstream effector enzymes, ultimately leading to apoptosis. Caspase-3, a pivotal executioner enzyme in this process, cleaves cytoskeletal proteins and is indicative of the onset of apoptosis [49]. The activity of caspase-3 and caspase-9 showed a decreasing trend during the ripening process (Figure 6a,b). Caspase-3 and caspase-9 activities in the ES group were significantly higher than those in the NS group throughout the process (*p* < 0.01). This finding suggests that the degradation process involving caspases was accelerated in the ES group relative to the NS group. This acceleration may be associated with the alterations in mitochondrial membrane permeability caused by Bcl-2 family proteins post-ES treatment, which subsequently affects the release of Cytochrome-C (Cyt-C) from the mitochondria. The binding of Cyt-C with caspase-9 forms an apoptosome [49], leading to the activation of caspase-9 and hastening the activation of downstream caspase-3 through the extrinsic pathway. This results in a higher degree of apoptosis in the ES group compared to the NS group.

It is noteworthy that necrosis represents a form of non-programmed cell death, distinct from apoptosis. Necroptosis is independent of caspase activation, and no apoptotic enzymes participate in the necrosis process [50]. This study documented changes in the activation of caspase-9 and caspase-3, indicating that apoptotic enzymes were triggered under ES conditions. This observation further substantiates that during the processing period, dry-cured pork loin experiences cell death predominantly through the mechanism of apoptosis.

### 3.8. HE Stain

**Figure 7:**
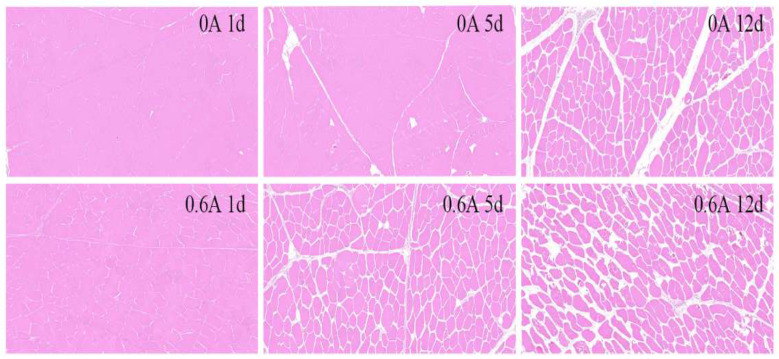
Photo of HE staining during ripening of dry-cured pork loin.

At day 1 (Figure 7), muscle fibers in both groups were closely packed, exhibiting a relatively intact muscle cell structure. The shape, size, and width of the muscle cells were uniform, and the intercellular spaces were minimal. As the ripening period extended, there was a significant increase in cellular spaces in both groups, accompanied by the appearance of wrinkling in the myocytes, which are characteristic signs of apoptosis. An enlargement of myocyte spaces during ripening has also been documented [51]. By day 5, the myocyte space in the NS group showed only a slight change. In contrast, the muscle fibers in the ES group began to swell, the cells became wrinkled and diminished in size, and the intercellular gaps widened. As ripening progressed, the muscle cell structure in the NS group underwent substantial changes by day 12. However, the ES group exhibited more pronounced wrinkling compared to the NS group, with the majority of cells appearing to be ruptured.

### 3.9. Apoptosis Rate

**Figure 8:**
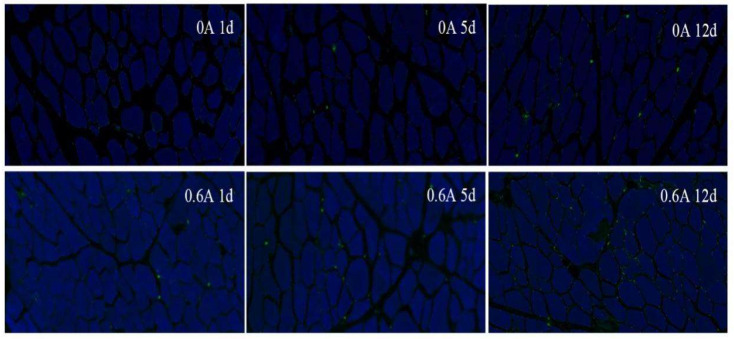
Effect of ES on TUNEL apoptotic nuclei changes during ripening of dry-cured pork loin.

**Figure 9:**
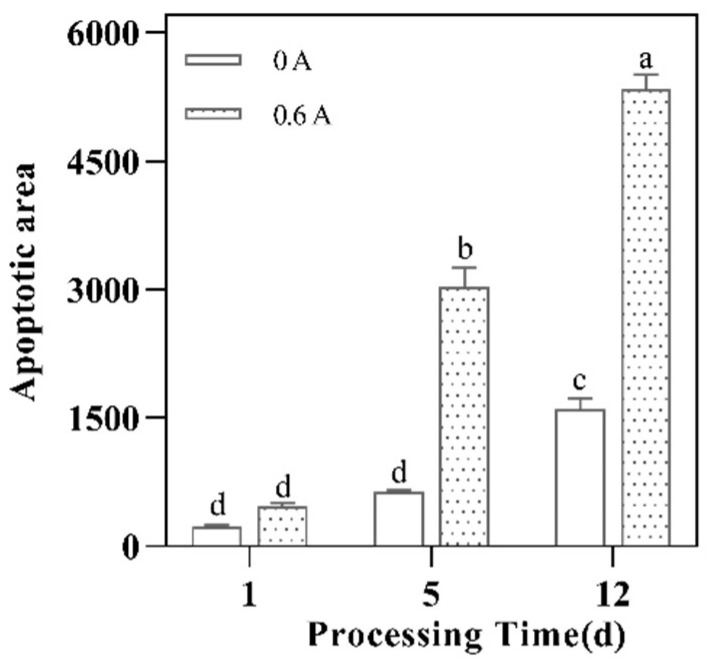
Effect of ES on the apoptotic area during ripening of dry-cured pork loin. ^a–d^ means the significant differences (*p* < 0.05).

To more accurately reflect the situation of apoptotic cells, the apoptotic cell area changes were used to reflect the cell apoptosis rate. The green fluorescence of each visual field picture is for apoptotic cells, and the blue fluorescence is for all cells. Both groups had very little apoptosis, and the number of green fluorescence increased, and the number of apoptotic cells gradually increased (Figure 8). At comparable time points, the apoptotic area in the ES group was approximately 2, 5, and 3 times that of the control group, respectively. Statistically significant differences were found between the ES and NS groups, with the apoptotic area in the ES group being markedly higher (*p* < 0.001) (Figure 9). The ES group displayed a significantly higher apoptotic rate compared to the NS group, which is indicative of increased degradation of caspase-9 and caspase-3. These findings corroborate the hypothesis that electrostimulation enhances cellular apoptosis.

### 3.10. Analysis of Potential Relationships between Heat Shock Proteins, Apoptosis, and Quality of Dry-Cured Pork Loin

**Figure 10:**
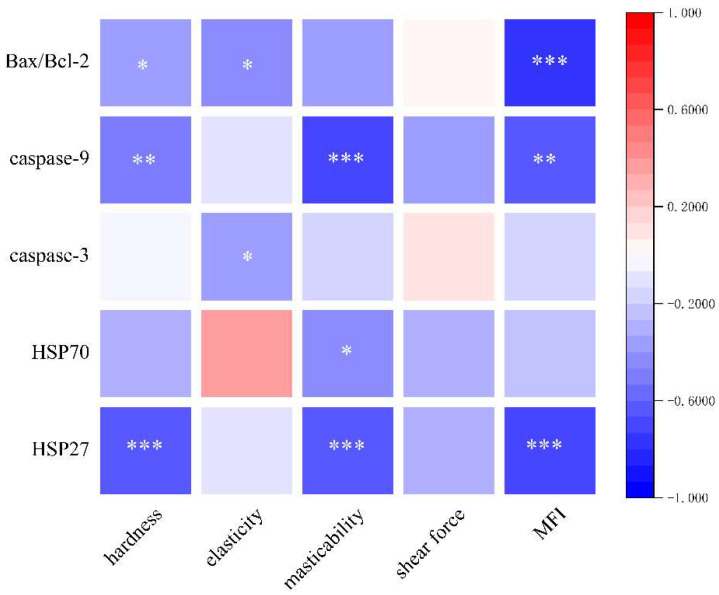
Analysis of potential relationships between HSP27, HSP70, apoptosis, and quality of dry-cured pork loin (* *p* < 0.05; ** *p* < 0.01; and *** *p* < 0.001).

A correlation analysis was conducted to evaluate the potential relationships between the expression of HSPs, apoptotic markers, and the quality attributes of dry-cured pork loin (Figure 10). HSP27 expression showed a significant positive correlation with hardness, machinability, and the MFI (*p* < 0.001). Additionally, HSP70 expression was positively correlated with the mastication properties of the meat (*p* < 0.05). The activity of caspase-3 demonstrated a significant positive correlation with elasticity (*p* < 0.05), and caspase-9 activity was correlated with hardness, masticability, and the MFI (*p* < 0.01). Furthermore, the ratio of Bax to Bcl-2, an indicator of apoptotic tendency, also exhibited significant correlations with hardness, elasticity (*p* < 0.05), and the MFI (*p* < 0.001). These findings suggest that the down-regulation of HSP expression and the activation of apoptotic pathways by ES indirectly impact the final quality of dry-cured pork loin. The modulation of these proteins and enzymes likely contributes to the observed changes in meat texture and tenderness.

HSPs are known to influence the organoleptic properties and characteristics of meat. In particular, HSP27 and its degradation products have been reported to account for 91% of the variance in tenderness [52]. However, the relationship between HSPs and meat tenderness is not uniformly documented in the literature. Ding et al. [53] observed a negative correlation between HSP27 expression and meat tenderness, while Zapata et al. [54] suggested that HSP27, involved in actin protection, exhibits a positive correlation with tenderness. The consensus among many researchers is that HSPs may exert an inhibitory effect on the enhancement of meat tenderness. The coordinated regulation of HSP27 and αβ-crystallin promotes the degradation of actin and myosin due to the chaperone effect of HSPs, thereby improving meat tenderness [55]. ES may induce structural damage to HSP27 and HSP70 by applying electrical currents to the tissues, leading to down-regulated expression levels. The loss of their chaperone function results in the cells losing their protective effect, culminating in myofibril degradation.

HSPs also play a crucial role in inhibiting the apoptotic pathway by interacting with key apoptotic proteins, thus preventing the typical apoptotic changes that occur during the late stages of apoptosis [56]. High expression levels of HSPs in post-mortem muscle may potentially prevent apoptosis and delay protein degradation. In the mitochondrial apoptosis pathway, Cyt-C is released from the mitochondria into the cytoplasm, where it binds to Apaf-1 and ATP, recruiting Procaspase-9 to form apoptotic bodies. This process activates caspase-3, initiating the caspase cascade and ultimately leading to apoptosis. HSPs inhibit the formation of these apoptotic bodies by Cyt-C, thereby hindering the caspase cascade reaction [13]. They are implicated in the regulation of caspase activity, modulation of apoptotic signaling upstream and downstream of the mitochondria, and control of the release of apoptotic molecules at the mitochondrial level [57].

Reduced expression of HSPs stimulates caspase activation, leading to enhanced degradation of myofibrillar and sarcomeric proteins. This degradation disrupts the structure of myofibrillar proteins, increasing the MFI value, reducing shear force, and enhancing meat tenderness [58]. Our research indicates that ES down-regulates the expression of HSP27 and HSP70 while accelerating the degradation of caspase-3 and caspase-9, and significantly elevating the Bax/Bcl-2 ratio. This sequence of molecular events induces an earlier onset of apoptosis, which in turn promotes the maturation and tenderness of dry-cured pork loin. It appears that the suppression of HSP27 and HSP70 by ES is intricately linked to the activation of apoptotic pathways.

## 4. Conclusions

Apoptosis plays an integral role throughout the production of dry-cured meat, with its presence being particularly pronounced during the curing stage. The implementation of ES during the pivotal transition from muscle to meat has been observed to significantly reduce shear force and alter muscle fiber structure. These changes indicate a marked enhancement in meat tenderness. Thus, a comprehensive understanding of apoptotic pathways could potentially be harnessed to utilize ES for accelerating this natural cellular adjustment process.

In this study, the roles of HSP27 and HSP70 have emerged as significant regulatory factors within the dry-curing process. Our findings suggest that ES accelerates the degradation of caspase-3 by intensifying mitochondrial oxidative damage and significantly promoting the release of pro-apoptotic factors, such as Bax. These mechanisms precipitate the premature triggering of apoptotic pathways, which are advantageous for protein breakdown. This acceleration could potentially enhance meat quality.

During the processing of cured meat, mitochondrial apoptotic pathways are also at play, and the quality and ripening of the meat can be effectively influenced through precise regulation by ES. Although further in-depth research is required to explore methods for shortening the production cycle and enhancing the quality of dry-cured meat products, the impact of ES during the processing stage on muscle fibers, mitochondrial apoptosis, and the expression of heat shock proteins offers new perspectives. These insights contribute to our understanding of the regulation of these meat quality attributes.

## Figures and Tables

**Table 1:** Effect of ES on pH, texture, and tenderness during ripening of dry-cured pork loin.

	Group	Processing Time (d)				
		1	5	12	20	30
pH	0 A	5.47 ± 0.01 ^g^	5.53 ± 0.03 ^f^	5.63 ± 0.01 ^d^	5.92 ± 0.01 ^bc^	6.10 ± 0.01 ^a^
	0.6 A	5.45 ± 0.01 ^g^	5.46 ± 0.00 ^g^	5.56 ± 0.00 ^e^	5.90 ± 0.01 ^c^	5.93 ± 0.01 ^b^
Hardness (gf)	0 A	79.5 ± 5.04 ^d^	36.8 ± 0.69 ^f^	82.9 ± 4.49 ^d^	111.5 ± 1.29 ^c^	3984.1 ± 269.53 ^a^
	0.6 A	76.1 ± 6.33 ^d^	29.8 ± 2.69 ^f^	56.1 ± 3.96 ^e^	77.2 ± 2.26 ^d^	1533.6 ± 211.57 ^b^
Elasticity (mm)	0 A	0.3 ± 0.02 ^g^	0.7 ± 0.02 ^ef^	0.7 ± 0.02 ^de^	0.8 ± 0.02 ^bc^	0.6 ± 0.03 ^f^
	0.6 A	0.3 ± 0.03 ^g^	0.8 ± 0.03 ^cd^	0.9 ± 0.02 ^ab^	0.9 ± 0.03 ^a^	0.7 ± 0.03 ^cd^
Masticability (mJ)	0 A	1.8 ± 0.07 ^g^	28.4 ± 3.44 ^e^	52.5 ± 3.63 ^d^	71.2 ± 2.21 ^c^	1460.0 ± 83.81 ^a^
	0.6 A	0.9 ± 0.08 ^g^	13.0 ± 1.48 ^f^	28.7 ± 2.60 ^e^	50.8 ± 5.93 ^d^	749.3 ± 42.88 ^b^
Shear force (N)	0 A	58.4 ± 2.44 ^d^	35.9 ± 1.53 ^ef^	47.4 ± 2.46 ^de^	107.1 ± 3.25 ^c^	210.3 ± 5.96 ^a^
	0.6 A	53.1 ± 4.00 ^d^	28.7 ± 2.69 ^f^	36.1 ± 0.96 ^ef^	63.3 ± 8.88 ^d^	140.7 ± 5.92 ^b^
MFI	0 A	27.9 ± 0.48 ^e^	50.8 ± 1.04 ^b^	44.2 ± 0.45 ^c^	22.3 ± 0.44 ^f^	18.6 ± 0.09 ^g^
	0.6 A	33.0 ± 0.52 ^d^	68.6 ± 1.94 ^a^	52.3 ± 0.40 ^b^	27.6 ± 0.12 ^e^	22.4 ± 0.21 ^f^

^a–g^ means the significant differences (*p* < 0.05) in the sample under different treatment conditions and processing times.

## Data Availability

The original contributions presented in the study are included in the article, further inquiries can be directed to the corresponding author.
